# Practical synthesis of *N*-(di-*n*-butylamino)methylene-protected 2-aminopurine riboside phosphoramidite for RNA solid-phase synthesis

**DOI:** 10.1007/s00706-019-02502-7

**Published:** 2019-10-11

**Authors:** Eva Neuner, Ronald Micura

**Affiliations:** Institute of Organic Chemistry, Leopold-Franzens University, Innrain 80-82, Innsbruck, Austria

**Keywords:** Nucleoside modification, Oligonucleotides, Fluorescence, 2ApFold, Ribozymes, Riboswitches

## Abstract

**Electronic supplementary material:**

The online version of this article (10.1007/s00706-019-02502-7) contains supplementary material, which is available to authorized users.

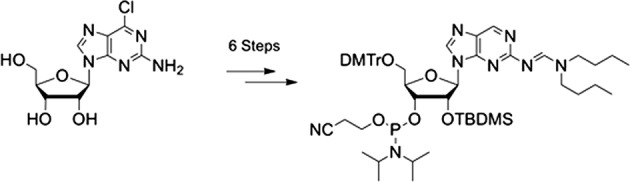

## Introduction

The nucleobase analog 2-aminopurine (Ap) is widely used as minimally invasive fluorescent reporter in RNA for the investigation of folding and ligand–RNA interactions [1–5]. Utilization of this label requires its site-specific incorporation into the RNA of interest which is generally achieved by RNA solid-phase synthesis based on appropriately functionalized building blocks.

Fox et al. [6] reported the first synthesis of 2-aminopurine riboside starting with the thiation of guanosine with phosphorus pentasulfide followed by the reduction with Raney nickel. Another synthetic route was published in the same year by Schaeffer et al. [7] utilizing the condensation of chloromercuri-2-benzamidopurine with 2,3,5-tri-*O*-benzoylribofuranosyl chloride followed by deprotection of the hydroxyl functions. Alternatively, Nair et al. [8] introduced a photochemical reduction of 6-chloro-2-aminopurine riboside to the corresponding 2-aminopurine nucleoside.

The first incorporation of 2-aminopurine into DNA oligomers, was reported by Eritja et al. [9] using the phosphotriester method. The first synthesis of a phosphor(III) amidite of 2-aminopurine 2′-deoxyriboside and its incorporation into DNA was described by McLaughlin et al. [10]. They obtained the nucleoside from the precursor of 6-hydrazino-2-aminopurine 2′-deoxyriboside in the presence of AgO_2_. Several reports on the synthesis of 2-aminopurine 2′-deoxyriboside phosphoramidite and its incorporation into DNA oligomers followed. Schmidt et al. [11] reduced the protected 6-chloro-2-aminopurine riboside with tri-*n*-butyltin hydride and azobis(isobutyronitril) (AIBN) to obtain the desired 2-aminopurine 2′-deoxyriboside while Fujimoto et al. [12] prepared the Ap phosphoramidite starting with the reduction of 6-thioguanosine using Raney nickel and subsequent deoxygenation of the 2′-OH via phenyl thiocarbonate formation and treatment with tri-*n*-butyltin hydride and AIBN. Parel et al. [13] obtained 2-aminopurine 2′-deoxyriboside via glycosylation as had been previously shown for glucopyranosyl-2-aminopurine nucleosides by Garner et al. [14].

The incorporation of 2-aminopurine into DNA was soon complemented with reports on 2-aminopurine riboside phosphoramidites for RNA synthesis by Doudna et al. [15] as well as Santalucia et al. [16]. The key step in the first path involved the reduction of 6-thioguanosine with Raney nickel whereas the second utilized the synthetic conception from McLaughlin et al. [10]. The exocyclic amine was protected with a benzoyl and isobutyryl group, respectively, followed by 2′-*O*-*tert*-butyldimethylsilyl (TBDMS) and 2′-*O*-tetrahydropyranyl protection, respectively, to furnish the corresponding 2-aminopurine riboside phosphoramidites. A similar synthesis was reported by Tuschl et al. [17] for the investigation of hammerhead ribozyme activity. Höbartner and co-workers [18] synthesized the 2-aminopurine phosphoramidite containing a 2′-*O*-TOM protecting group. Zagórowska et al. [19] obtained the 2-aminopurine riboside phosphoramidite by reduction of the triacetylated 6-chloro-2-aminopurine riboside with hydrogen and palladium on carbon followed by standard functionalization for TBDMS RNA chemistry. Buchini et al. [20] and Peacock et al. [21] also reported the hydrogenation of 6-chloro-2-aminopurine as key step for the generation of 2′-*O*-aminoethyl or N^2^-alkylated 2-aminopurine riboside phosphoramidites. Koshkin [22] employed a similar strategy using palladium hydroxide on carbon and ammonium formate to hydrogenate the 6-chloro-2-aminopurine in locked nucleic acids (LNA) nucleosides in high yields.

Here, we introduce a robust synthetic path for a 2-aminopurine riboside phosphoramidite, starting from inexpensive 6-chloro-2-aminopurine riboside. In the target compound, the N^2^ functionality is masked with the *N*-(di-*n*-butylamino)methylene group [23, 24]. For 2-aminopurine riboside building blocks, this protection is considered advantageous over previously described *N*-(dimethylamino)methylene [15, 22] or acyl protection [10–13, 18, 19] patterns, needed for the fine-tuned deprotection conditions to achieve large synthetic RNAs.

## Results and discussion

**Scheme 1:**
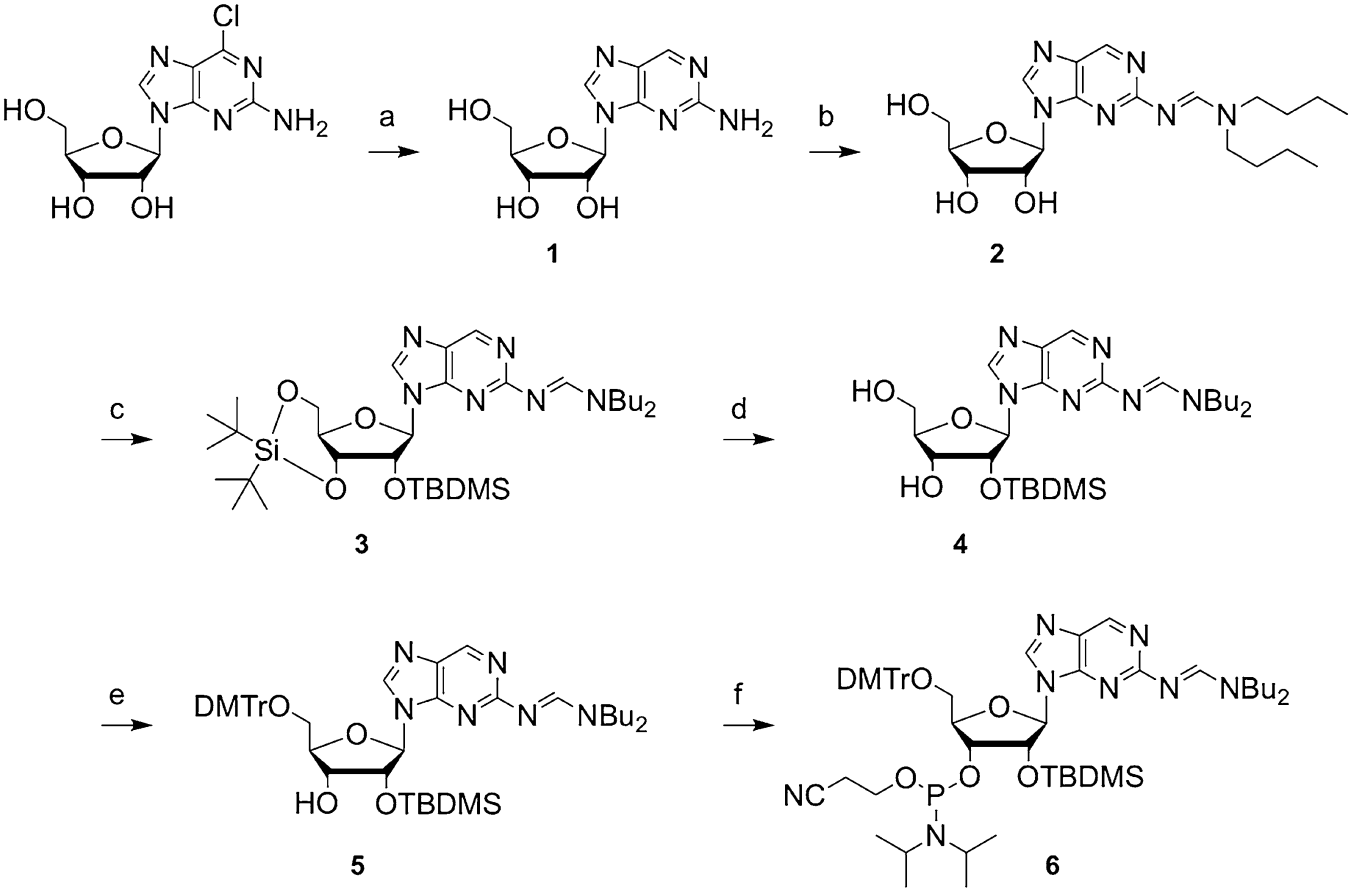
Reaction conditions: **a** 0.3 equiv Pd(OH)_2_/C, 10 equiv ammonium formate in CH_3_OH:dioxane (1:1), 1 h, reflux, quantitative; **b** 3 equiv Bu_2_NCH(OCH_3_)_2_ in CH_3_OH, 2 d, room temperature to 50 °C, 83%; **c** i) 1.1 equiv di-*tert*-butylsilyl bis(trifluoromethanesulfonate) ((tBu)_2_Si(OTf)_2_) in DMF, 30 min, 0 °C, ii) 5 equiv imidazole in DMF, 15 min at 0 °C, then 15 min at room temperature, iii) 1.3 equiv *tert*-butyldimethylsilyl chloride (TBDMSCl) in DMF, 2 h, 60 °C, 85%; **d** 3.8 equiv HF-pyridine in CH_2_Cl_2_, 0 °C, 2 h, 61%; **e** 1.2 equiv 4,4′-dimethoxytrityl chloride (DMTrCl) in pyridine, 3 h, room temperature, 90%; **f** 0.6 equiv 1-methylimidazole, 7 equiv *sym*-collidine, 2.5 equiv 2-cyanoethyl *N*,*N*-diisopropylchlorophosphoramidite (CepCl) in THF, room temperature, 1.5 h, 84%

Our synthetic route to the functionalized 2-aminopurine riboside phosphoramidite **6** starts with the reduction of the commercially available 2-amino-6-chloropurine riboside using Pearlman′s catalyst (Pd(OH)_2_/C) and ammonium formate to yield compound **1** (Scheme 1). The exocyclic 2-amino function was selectively protected by treatment with *N*,*N*-dibutylformamide dimethyl acetal (DBFDMA) [25–29] producing nucleoside derivative **2**. In the next step, the 5′ and 3′ hydroxyl groups were simultaneously protected by reaction with di-*tert*-butylsilyl bis(trifluoromethanesulfonate) ((*t*Bu)_2_Si(OTf)_2_) [30, 31], followed by silylation of the 2′-hydroxyl group with *tert*-butyldimethylsilyl chloride (TBDMSCl) to give compound **3**. The 5′ and 3′ hydroxyl protection clamp was then selectively removed with a solution of HF in pyridine to yield compound **4**. The functionalization of the 5′ hydroxyl group with 4,4′-dimethoxytrityl chloride was achieved under standard conditions to give compound **5** in high yields. In the final step, the phosphoramidite **6** was generated by treatment with 2-cyanoethyl *N*,*N*-diisopropylchlorophosphoramidite (CepCl) in the presence of 2,4,6-trimethylpyridine and *N*-methylimidazole in THF, conditions that we applied to avoid migration of the 2′-*O*-TBDMS group during preparation of the ribonucleoside phosphoramidite [32]. Starting from 2-amino-6-chloropurine riboside, the target compound **6** was synthesized in six steps, with five chromatographic purifications and an overall yield of 33%.

We note that the *N*-(di-*n*-butylamino)methylene-protected 2-aminopurine phosphoramidite **6** is coupled under standard conditions for RNA solid-phase synthesis with yields comparable to the 2′-*O*-TBDMS standard nucleoside building blocks. Oligoribonucleotides are deprotected under typical deprotection conditions (e.g. a 1:1 mixture of 40% aqueous methylamine and 30% aqueous ammonia (AMA) for 4 h at room temperature or 45 min at 65 °C) (Supporting Fig. S1). Also ultramild conditions, such as 4 h at RT with 0.05 M potassium carbonate in methanol or 2 h at RT with ammonium hydroxide can be applied, provided acetic anhydride is replaced by phenoxyacetic anhydride for capping, and phenoxyacetyl-protected phosphoramidite building blocks are used in combination.

2-Aminopurine is a fluorescent isomer of adenine and an important nucleobase modification for the elucidation of structural and functional properties of nucleic acids. In particular, several laboratories apply an approach termed 2-aminopurine-based RNA folding analysis (2ApFold) [2, 3, 33] which has been developed to provide insights into the folding and folding dynamics of RNA. This approach relies on synthetic RNAs with a single, strategically positioned 2-aminopurine which substitutes a nucleobase under minimal steric interference and preservation of existing H-bond and stacking patterns. Thereby, the selection of suitable Ap positions is either based on the three-dimensional structure (if available) or on SHAPE probing [3, 34, 35]. Hence, the conformational rearrangements of the Ap-labeled RNA in response to external stimuli (such as Mg^2+^ cations or low-molecular weight compounds that bind RNA) are analyzed by pursuing the Ap fluorescence intensity signal, revealing the underlying kinetic and thermodynamic parameters. Beside the 2ApFold approach, a series of recent reports is available that utilize 2-aminopurine fluorescence spectroscopy to determine the cleavage kinetics of small nucleolytic ribozymes [36–38].

## Conclusion

We have developed a convenient 6-step synthetic route toward an amidine-protected 2-aminopurine riboside phosphoramidite for RNA solid-phase synthesis, starting from inexpensive 2-amino-6-chloropurine riboside. The two key features of the path are first, hydrogenolysis of the 6-chloropurine derivative which we experienced advantageous in terms of reproducibility over previously described routes that involved reduction of 6-thioguanosine derivatives, and second, N^2^ protection by the *N*-(di-*n*-butylamino)methylene group which allows the application of optimized, mild deprotection conditions required for the preparation of large synthetic RNA with more than 50 nucleotides.

## Experimental

Reagents were purchased in the highest available quality from commercial suppliers (Sigma-Aldrich, abcr, Carbosynth) and used without further purification. Moisture-sensitive reactions were carried out under argon atmosphere. ^1^H and ^13^C spectra were recorded on a Bruker DRX 400 MHz spectrometer. Chemical shifts (*δ*) are reported relative to tetramethylsilane (TMS) referenced to the residual proton signal of the deuterated solvent (DMSO-d_6_: 2.50 ppm for ^1^H spectra and 39.52 ppm for ^13^C spectra; CDCl_3_: 7.26 ppm for ^1^H spectra and 77.16 ppm for ^13^C spectra). Signal assignments are based on ^1^H-^1^H-COSY and ^1^H-^13^C-HSQC experiments. MS experiments were performed on a Thermo Scientific Q Exactive Orbitrap with an electrospray ion source in the positive mode. Reaction control was performed via analytical thin-layer chromatography (TLC, Macherey–Nagel) with fluorescent indicator. Column chromatography was carried out on silica gel 60 (70–230 mesh).

### 2-Amino-9-(*β*-d-ribofuranosyl)purine (1, C_10_H_13_N_5_O_4_)

2-Amino-6-chloropurine riboside (877 mg, 2.91 mmol) was dissolved in 15 cm^3^ methanol:dioxane (1:1) and 612 mg palladium hydroxide on carbon (20 wt% loading, 0.872 mmol) and 1835 mg ammonium formate (29.1 mmol) were added. The mixture was refluxed for 1 h, cooled to room temperature, and filtered through a Celite pad. The solvents were evaporated and the product was dried under high vacuum. No further purification was performed and a white solid was obtained in quantitative yield. HR-ESI–MS: *m*/*z* calculated for [C_10_H_14_N_5_O_4_]^+^ ([M+H]^+^): 268.1040, found 268.1025; ^1^H NMR (400 MHz, DMSO-d_6_): *δ* = 3.51–3.56 (m, 1H, H_b_C(5′)), 3.60–3.66 (m, 1H, H_a_C(5′)), 3.89–3.92 (m, 1H, HC(4′)), 4.11–4.14 (m, 1H, HC(3′)), 4.48–4.53 (m, 1H, HC(2′)), 5.06 (t, 1H, HO(5′)), 5.16 (d, 1H, HO(3′)), 5.44 (d, 1H, HO(2′)), 5.83 (d, 1H, HC(1′)), 6.55 (2H, H_2_N(2)), 8.30 (1H, HC(8)), 8.59 (1H, HC(6)) ppm; ^13^C NMR (100 MHz, DMSO-d_6_): *δ* = 61.83 (C(5′)), 70.87 (C(3′)), 73.90 (C(2′)), 85.78 (C(4′)), 86.66 (C(1′)), 141.27 (C(8)), 149.74 (C(8)) ppm.

### 2-[*N*-(Di-*n*-butylamino)methylene]amino-9-(*β*-d-ribofuranosyl)purine (2, C_19_H_30_N_6_O_4_)

Compound **1** (770 mg, 2.88 mmol) was dissolved in 5 cm^3^ methanol and 1758 mg *N*,*N*-dibutylformamide dimethyl acetal (8.64 mmol) was added dropwise. The solution was stirred at room temperature for 2 days and then heated to 50 °C for 4 h. The solvents were evaporated and the crude product was purified by column chromatography on silica gel (methanol:dichloromethane 1:99–10:90) to yield 969 mg (83%) of **2** as white foam. TLC (methanol:dichloromethane 5:95): *R*_f_ = 0.25; HR-ESI–MS: *m*/*z* calculated for [C_19_H_31_N_6_O_4_]^+^ ([M+H]^+^): 407.2401, found 407.2387; ^1^H NMR (400 MHz, CDCl_3_): *δ* = 0.83–0.94 (m, 6H, 2× H_3_C), 1.29–1.34 (m, 4 H, 2× H_2_C), 1.55–1.63 (m, 4H, 2× H_2_C), 3.27–3.30 (m, 2H, H_2_CN), 3.34–3.56 (m, 2H, H_2_CN), 3.67–3.71 (m, 1H, H_b_C(5′)), 3.85–3.88 (m, 1H, H_a_C(5′)), 4.23 (1H, HC(4′)), 4.41 (d, 1H, HC(3′)), 4.99–5.02 (m, 1H, HC(2′)), 5.80 (d, 1H, HC(1′)), 7.88 (1H, HC), 8.42 (1H, HC(8)), 8.59 (1H, HC(6)) ppm; ^13^C NMR (100 MHz, CDCl_3_): *δ* = 13.87 (C(nbf)), 19.82 (C(nbf)), 29.15 (C(nbf)), 31.13 (C(nbf)), 45.60 (C(nbf)), 52.15 (C(nbf)), 62.96 (C(5′)), 72.34 (C(3′)), 73.16 (C(2′)), 87.06 (C(4′)), 90.46 (C(1′)), 143.38 (C(nbf)), 149.45 (C(6)), 158.26 (C(8)) ppm.

### 2-[*N*-(Di-*n*-butylamino)methylene]amino-9-(*β*-d-ribofuranosyl)-2′-*O*-(*tert*-butyldimethylsilyl)-3′,5′-*O*-(di-*tert*-butylsilylene)purine (3, C_33_H_60_N_6_O_4_Si_2_)

Compound **2** (495 mg, 1.23 mmol) was co-evaporated three times with dry pyridine, dried under high vacuum, and dissolved in 2 cm^3^ dry *N*,*N*-dimethylformamide in an ice bath. Di-*tert*-butylsilyl bis(trifluoromethanesulfonate) (590 mg, 1.34 mmol) was added dropwise over a period of 15 min and the reaction mixture was stirred at 0 °C for 30 min. Imidazole (419 mg, 6.15 mmol) was added and the mixture was stirred for 15 min at 0 °C and for 15 min at room temperature. Then, 241 mg *tert*-butyldimethylsiliyl chloride (1.59 mmol) was added and the solution was stirred at 60 °C for another 2 h. The mixture was diluted with dichloromethane, washed with brine, dried over sodium sulfate, and evaporated. The crude product was purified by column chromatography on silica gel (methanol:dichloromethane 0:100–2:98) to yield 695 mg (85%) of **3** as white foam. TLC (methanol:dichloromethane 5:95): *R*_f_ = 0.46; HR-ESI–MS: *m*/*z* calculated for [C_33_H_61_N_6_O_4_Si_2_]^+^ ([M+H]^+^): 661.4287, found 661.4269; ^1^H NMR (400 MHz, CDCl_3_): *δ* = 0.14–0.17 (d, 6H, 2× H_2_C(TBDMS)), 0.93 (9H, H_2_C(TBDMS)), 1.04–1.06 (24H, 8× H_3_C), 1.32–1.42 (m, 4H, 2× H_2_C), 1.60–1.68 (m, 4H, 2× H_2_C), 3.30–3.34 (t, 2H, H_2_CN), 3.58–3.63 (t, 2H, H_2_CN), 4.01–4.06 (m, 1H, H_b_C(5′)), 4.19–4.30 (m, 2H, HC(3′), HC(4′)), 4.49–4.52 (m, 1H, H_a_C(5′)), 4.55 (d, 1H, HC(2′)), 6.06 (1H, HC(1′)), 7.86 (1H, HC), 8.65 (1H, HC(8)), 8.90 (1H, HC(6)) ppm; ^13^C NMR (100 MHz, CDCl_3_): *δ* = − 4.78 (C(TBDMS)), − 4.15 (C(TBDMS)), 13.87 (C(TBDMS)), 14.07 (C(TBDMS)), 19.98 (C(nbf)), 26.04 (C(TBDMS)), 27.15 (Si(C(CH_3_)_3_)_2_), 27.43 (C(nbf)), 29.36 (C(nbf)), 31.37 (C(nbf)), 45.46 (C(nbf)), 51.99 (C(nbf)), 68.00 (C(5′)), 74.52 (C(4′)), 75.72 (C(3′)), 76.47 (C(2′)), 91.41 (C(1′)), 140.96 (C(nbf)), 150.01 (C(6)), 157.92 (C(8)) ppm.

### 2-[*N*-(Di-*n*-butylamino)methylene]amino-9-(*β*-d-ribofuranosyl)-2′-*O*-(*tert*-butyldimethylsilyl)purine (4, C_22_H_44_N_6_O_4_Si)

Compound **3** (695 mg, 1.05 mmol) was dissolved in 3 cm^3^ dichloromethane in an ice bath. Hydrogen fluoride in pyridine (8 M, 0.1 cm^3^, 3.99 mmol) was diluted with 0.6 cm^3^ cold pyridine and added dropwise to compound **3**. The reaction mixture was stirred at 0 °C for 2 h. Then, it was diluted with dichloromethane and saturated sodium bicarbonate was added. Stirring was continued until no more gas evolution was observed, the organic phase was washed twice more with saturated sodium bicarbonate, dried over sodium sulfate, and evaporated. The crude product was purified by column chromatography on silica gel (methanol:dichloromethane 0:100–3:97) to yield 332 mg (61%) of **4** as white foam. TLC (methanol:dichloromethane 5:95): *R*_f_ = 0.42; HR-ESI–MS: *m*/*z* calculated for [C_22_H_45_N_6_O_4_Si]^+^ ([M+H]^+^): 521.3266, found 521.3255; ^1^H NMR (400 MHz, CDCl_3_): *δ* = − 0.19 (3H, H_3_C(TBDMS)), 0.00 (3H, H_3_C(TBDMS)), 0.98 (9H, 3× H_3_C (TBDMS)), 1.11–1.15 (m, 6H, 2× H_3_C), 1.50–1.59 (m, 4H, 2× H_2_C), 1.76–1.82 (m, 4H, 2× H_2_C), 3.49–3.52 (m, 2H, H_2_CN), 3.66–3.89 (m, 2H, H_2_CN), 3.91–3.95 (m, 1H, H_b_C(5′)), 4.13–4.16 (m, 1H, H_a_C(5′)), 4.53 (2H, HC(3′), HC(4′)), 5.36–5.39 (t, 1H, HC(2′)), 5.91 (d, 1H, HC(1′)), 7.99 (1H, HC), 8.73 (1H, HC(8)), 9.10 (1H, HC(6)) ppm; ^13^C NMR (100 MHz, CDCl_3_): *δ* = − 5.30 (C(TBDMS)), − 5.19 (C(TBDMS)), 13.85 (C(TBDMS)), 13.99 (C(TBDMS)), 19.94 (C(nbf)), 20.26 (C(nbf)), 25.65 (C(TBDMS)), 29.30 (C(nbf)), 31.25 (C(nbf)), 45.48 (C(nbf)), 51.78 (C(nbf)), 63.40 (C(5′)), 73.02 (C(3′)), 74.06 (C(2′)), 87.61 (C(4′)), 91.08 (C(1′)), 143.17 (C(nbf)), 150.84 (C(6)), 158.01 (C(8)) ppm.

### 2-[*N*-(Di-*n*-butylamino)methylene]amino-9-(*β*-d-ribofuranosyl)-5′-*O*-(4,4′-dimethoxytrityl)-2′-*O*-(*tert*-butyldimethylsilyl)purine (5, C_46_H_62_N_6_O_6_Si)

Compound **4** (330 mg, 0.63 mmol) was co-evaporated three times with pyridine, dried under high vacuum for 1 h, and dissolved in 2 cm^3^ pyridine at room temperature. 4,4′-Dimethoxytrityl chloride (258 mg, 0.78 mmol) was dried under high vacuum for 1 h prior to its addition in 4 portions over 90 min to the solution. The reaction mixture was stirred for 4 h at room temperature until the starting material was fully consumed. Then, the solution was diluted with dichloromethane, washed with 5% citric acid and saturated sodium bicarbonate, dried over sodium sulfate, and evaporated. The crude product was purified by column chromatography on silica gel (methanol:dichloromethane 0:100–2:98) to yield 465 mg (90%) of **5** as white foam. TLC (methanol:dichloromethane 5:95): *R*_f_ = 0.46; HR-ESI–MS: *m*/*z* calculated for [C_46_H_63_N_6_O_6_Si]^+^ ([M+H]^+^): 823.4573, found 823.4547; ^1^H NMR (400 MHz, CDCl_3_): *δ* = − 0.15 (3H, H_3_C(TBDMS)), 0.00 (3H, H_3_C(TBDMS)), 0.84 (9H, 3× H_3_C (TBDMS)), 0.92–0.97 (m, 6H, 2× H_3_C), 1.31–1.40 (m, 4H, 2× H_2_C), 1.56–1.66 (m, 4H, 2× H_2_C), 3.28–3.32 (m, 2H, H_2_CN), 3.37–3.52 (m, 2H, H_2_C(5′)), 3.59–3.65 (m, 2H, H_2_CN), 3.78 (6H, 2× H_3_CO(DMT)), 4.22-4.24 (m, 1H, HC(4′)), 4.31–4.34 (m, 1H, HC(3′)), 4.75 (t, 1H, HC(2′)), 6.25 (d, 1H, HC(1′)), 6.81–6.83 (m, 4H, HC(DMT)), 7.31–7.44 (m, 9H, HC(DMT)), 8.09 (1H, HC), 8.66 (1H, HC(8)), 8.89 (1H, HC(6)) ppm; ^13^C NMR (100 MHz, CDCl_3_): *δ* = − 5.05 (C(TBDMS)), − 4.82 (C(TBDMS)), 13.88 (C(nbf)), 14.09 (C(nbf)), 19.94 (C(nbf)), 20.37 (C(nbf)), 25.73 (C(TBDMS)), 29.35 (C(nbf)), 31.30 (C(nbf)), 45.32 (C(nbf)), 51.82 (C(nbf)), 63.77 (C(5′)), 71.82 (C(3′)), 76.76 (C(2′)), 84.05 (C(4′)), 86.54 (C(1′)), 113.41 (C(DMT)), 127.19 (C(DMT)), 128.12 (C(DMT)), 128.24 (C(DMT)), 130.22 (C(DMT)), 141.04 (C(nbf)), 149.88 (C(6)), 158.09 (C(8)) ppm.

### 2-[*N*-(Di-*n*-butylamino)methylene]amino-9-(*β*-d-ribofuranosyl)-5′-*O*-(4,4′-dimethoxytrityl)-2′-*O*-(*tert*-butyldimethylsilyl)purine 3′-*O*-2-cyanoethyl-*N*,*N*-diisopropylphosphoramidite (6, C_55_H_79_N_8_O_7_PSi)

Compound **5** (100 mg, 0.12 mmol) was co-evaporated three times with dry pyridine, three times with dry toluene, and three times with dry tetrahydrofuran, and dried under high vacuum for 1 h. It was dissolved in 1 cm^3^ dry tetrahydrofuran and 5.9 mg 1-methylimidazole (0.07 mmol) and 102 mg 2,4,6-collidine (0.84 mmol) were added subsequently. Then, 71 mg 2-cyanoethyl *N*,*N*-diisopropylchlorophosphoramidite (0.3 mmol) was added dropwise and the solution was stirred at room temperature for 1.5 h. The reaction mixture was diluted with dichloromethane, washed with saturated sodium bicarbonate, and dried over sodium sulfate. The crude product was purified by column chromatography on silica gel (ethyl acetate:cyclohexane 3:7–4:6) to yield 103 mg (84%) of **6** as white foam. TLC (methanol:dichloromethane 3:97): *R*_*f*_ = 0.36 (both isomers); HR-ESI–MS: *m*/*z* calculated for [C_55_H_80_N_8_O_7_PSi]^+^ ([M+H]^+^): 1023.5651, found 1023.5619; ^1^H NMR (400 MHz, CDCl_3_): *δ* = − 0.163 (3H, H_3_C(TBDMS)), 0.00 (3H, H_3_C(TBDMS)), 0.77 (9H, H_3_C (TBDMS)), 0.92–0.96 (m, 6H, (H_3_C)_2_CHN), 1.02 (3H, H_3_C), 1.16–1.20 (m, 9H, (H_3_C)_2_CHN, H_3_C), 1.30–1.40 (m, 4H, 2× H_2_C), 1.57–1.68 (m, 4H, 2× H_2_C), 2.26–2.30 (m, 1H, HC(H_3_C)_2_N), 2.59–2.70 (m, 1H, HC(H_3_C)_2_N), 3.25–3.34 (m, 3H, H_2_CN, H_a_C(5′)), 3.44–3.48 (m, 1H, H_b_C(5′)), 3.34–3.63 (m, 6H, H_2_CN, H_2_CO, H_2_CN), 3.78 (6H, 2× H_3_CO(DMT)), 4.30–4.38 (m, 2H, HC(3′), HC(4′)), 4.73–4.78 (m, 1H, HC(2′)), 6.23–6.31 (dd, 1H, HC(1′)), 6.80–6.84 (m, 4H, HC(DMT)), 7.28–7.46 (m, 9H, HC(DMT)), 8.13–8.18 (1H, HC), 8.66 (1H, HC(8)), 8.88 (1H, HC(6)) ppm; ^31^P NMR (161 MHz, CDCl_3_): *δ* = 149.22, 150.92 ppm.

## Electronic supplementary material

Below is the link to the electronic supplementary material.

Supplementary material 1 (PDF 980 kb)
