# Supplementary material for: Practical synthesis of N-(di-n-butylamino)methylene-protected 2-aminopurine riboside phosphoramidite for RNA solid-phase synthesis
Source: Monatsh Chem. 2019 Oct 11;150(11):1941–6. doi: 10.1007/s00706-019-02502-7 (PMC6936340; doi:10.1007/s00706-019-02502-7)
Supplement: Supplementary file 1 — Supplementary material 1 (PDF 980 kb) [file 706_2019_2502_MOESM1_ESM.pdf]

Supporting Information

to

Practical synthesis of *N*-(di-*n*-butylamino)methylene-protected 2-aminopurine riboside phosphoramidite for RNA solid-phase synthesis

Eva Neuner[1] ● Ronald Micura[1]

[1]Institute of Organic Chemistry and Center for Molecular Biosciences,
University of Innsbruck, Austria

Contents

1. NMR spectra of compound **1** 2
2. NMR spectra of compound **2** 3
3. NMR spectra of compound **3** 4
4. NMR spectra of compound **4** 5
5. NMR spectra of compound **5** 6
6. NMR spectra of compound **6** 7

7. Supporting Figure 1 9

NMR spectra of compound 1

^{1}H-NMR (400 MHz, d_6-DMSO)

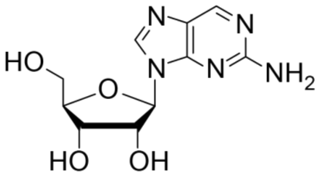

1

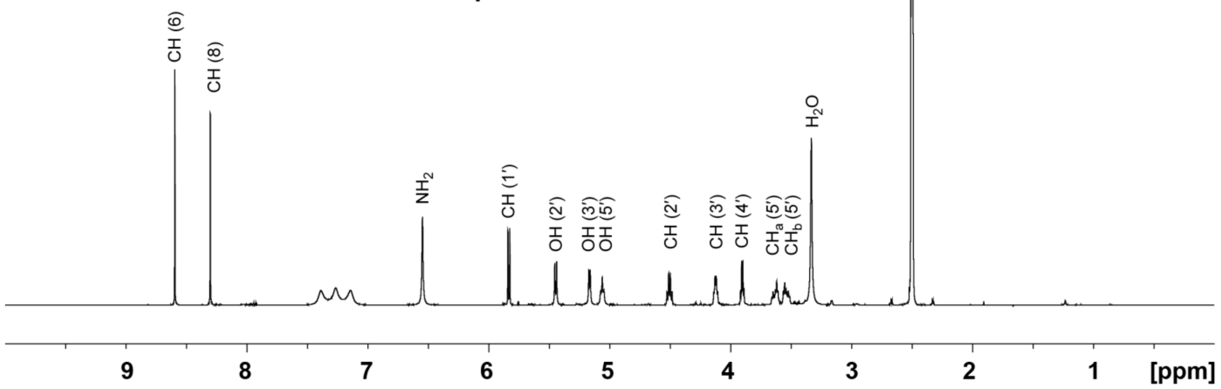

^{13}C-NMR (100 MHz, d_6-DMSO)

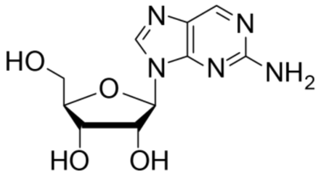

1

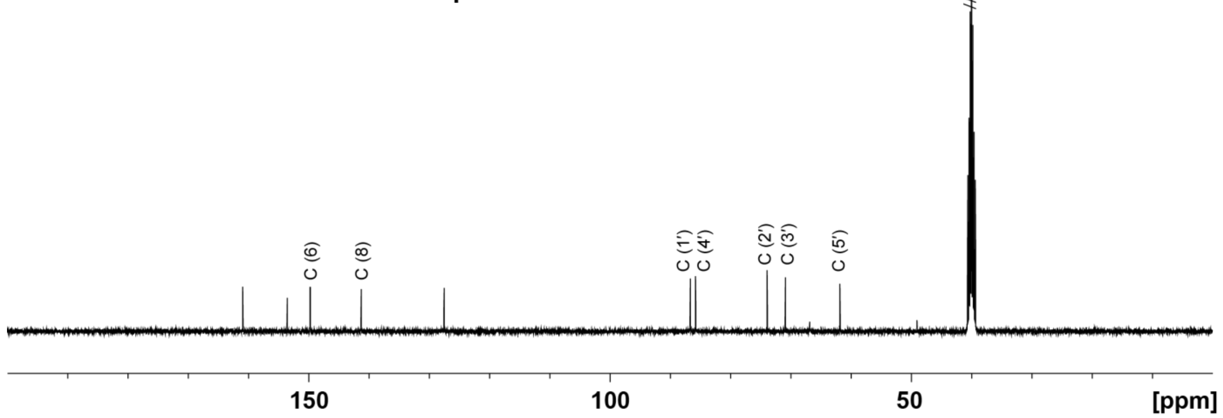

NMR spectra of compound 2

^{1}H-NMR (400 MHz, $CDCl_3$)

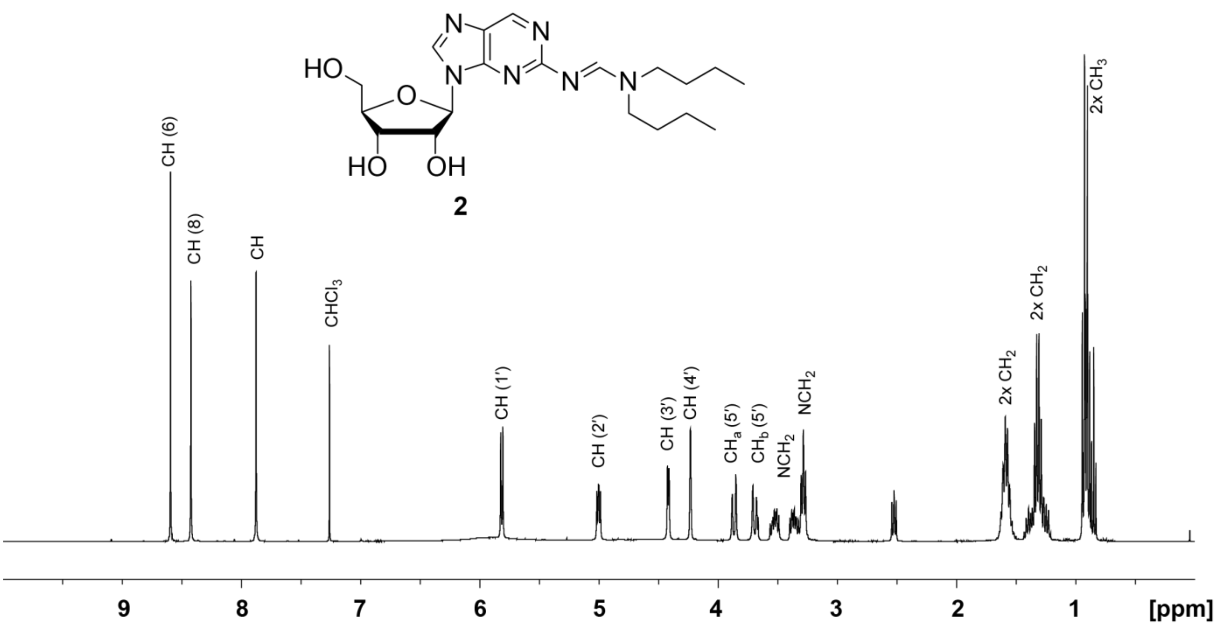

^{13}C-NMR (100 MHz, $CDCl_3$)

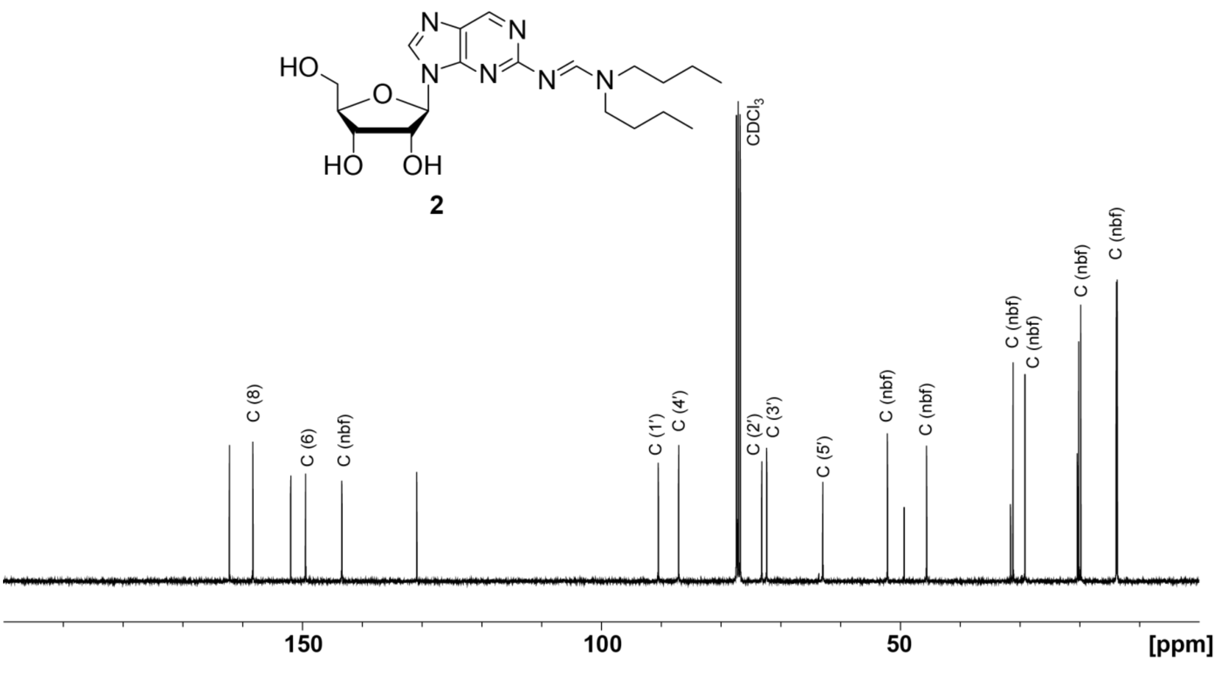

NMR spectra of compound 3

^{1}H-NMR (400 MHz, $CDCl_3$)

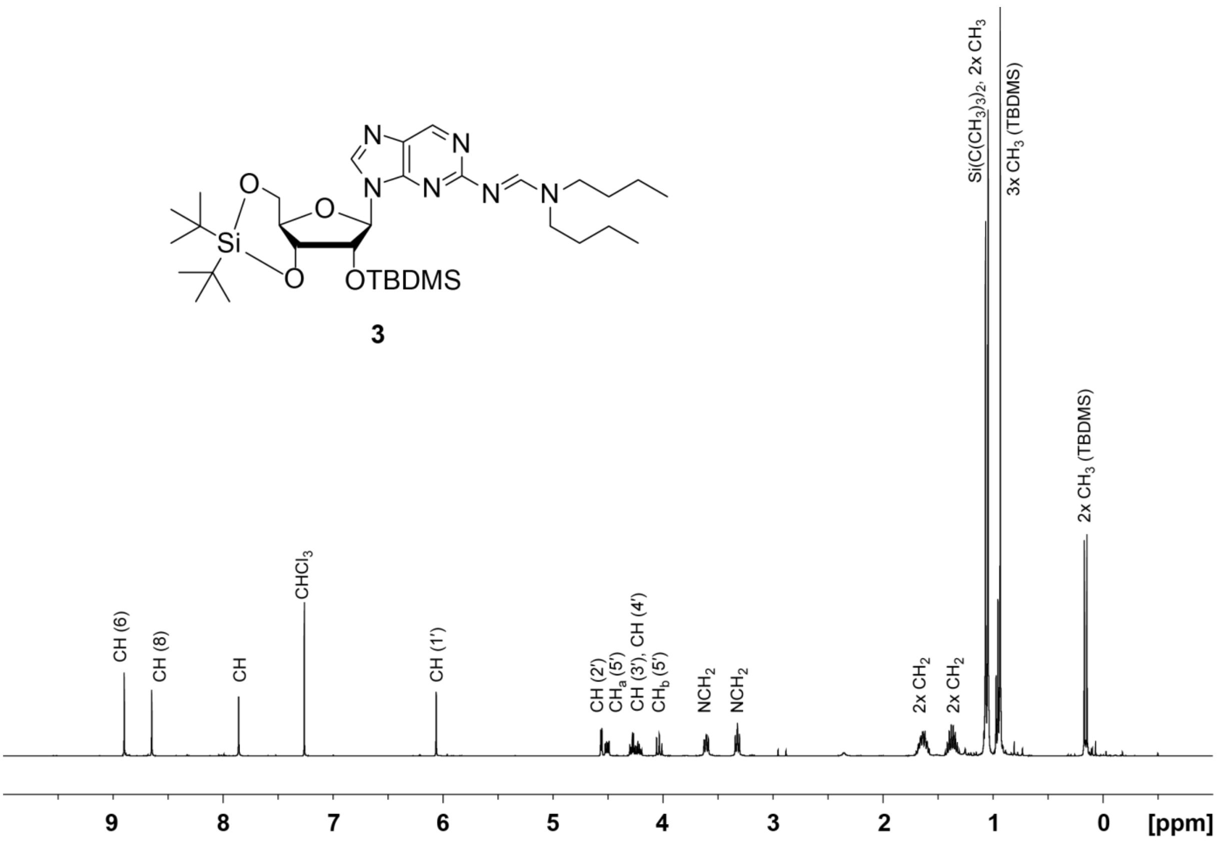

^{13}C-NMR (100 MHz, $CDCl_3$)

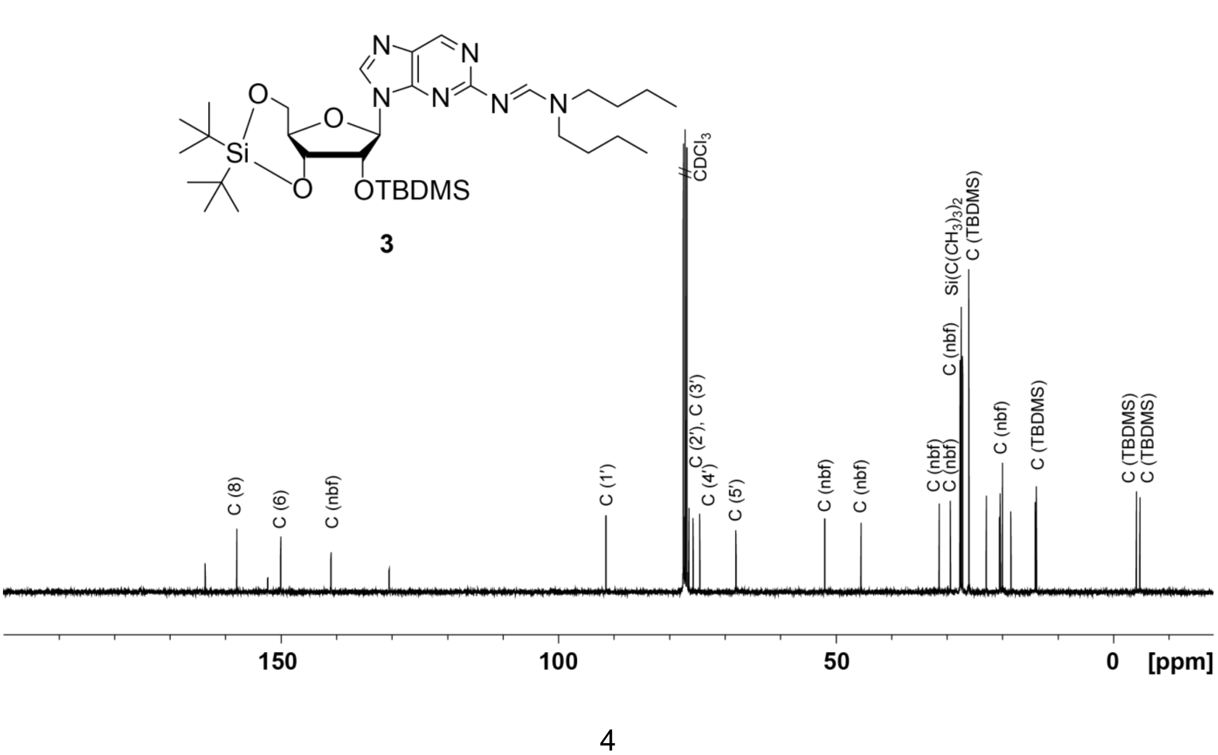

NMR spectra of compound 4

^{1}H-NMR (400 MHz, $CDCl_3$)

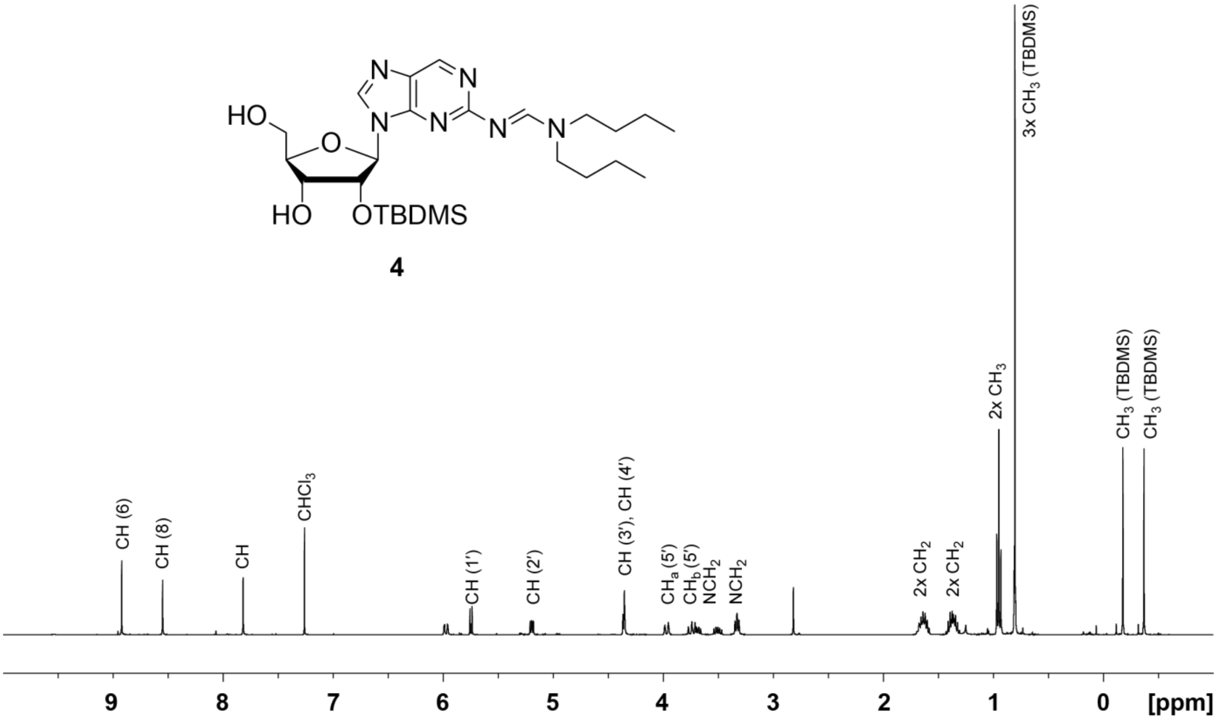

^{13}C-NMR (100 MHz, $CDCl_3$)

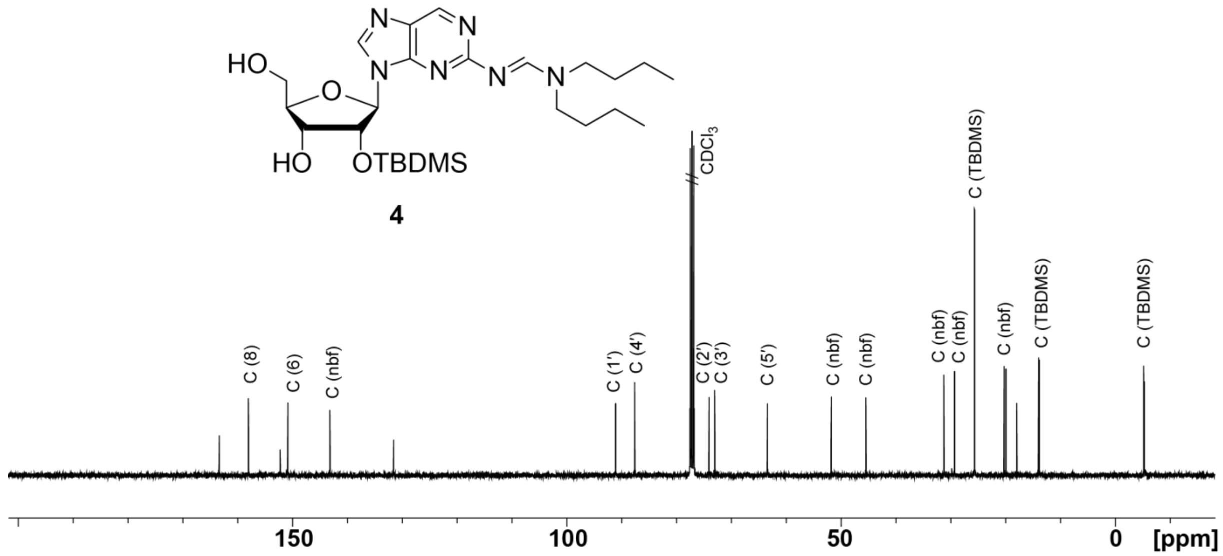

NMR spectra of compound 5

^{1}H-NMR (400 MHz, $CDCl_3$)

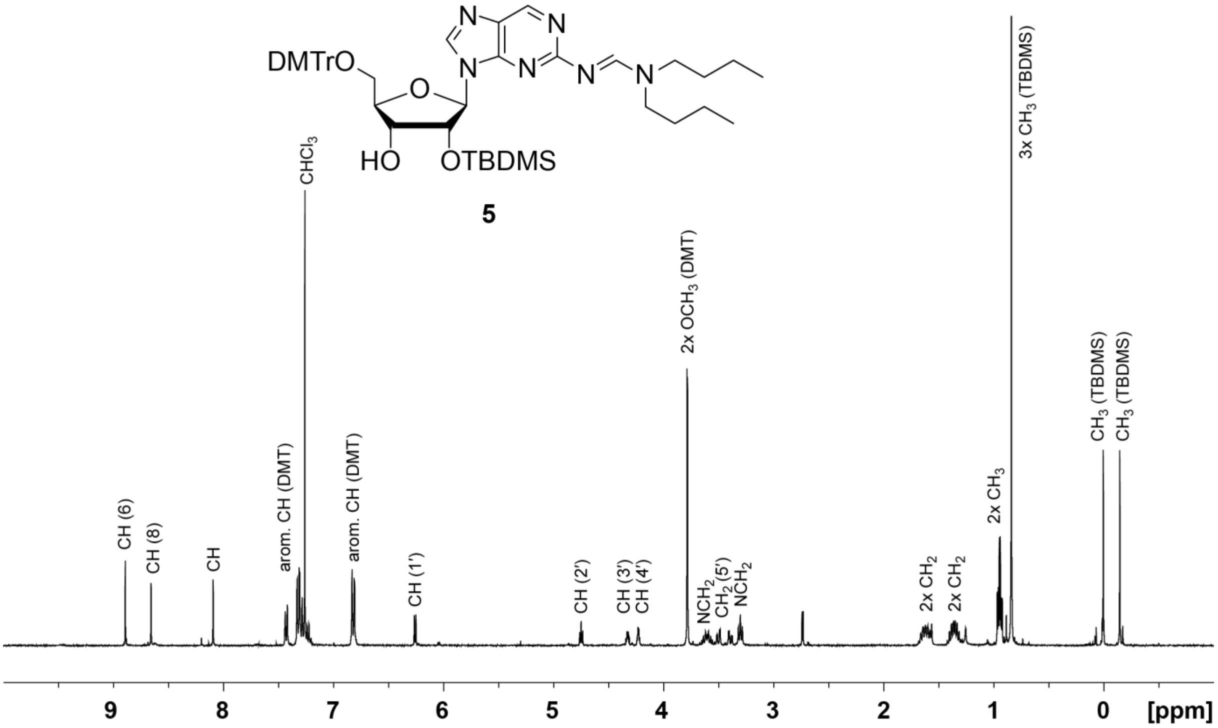

^{13}C-NMR (100 MHz, $CDCl_3$)

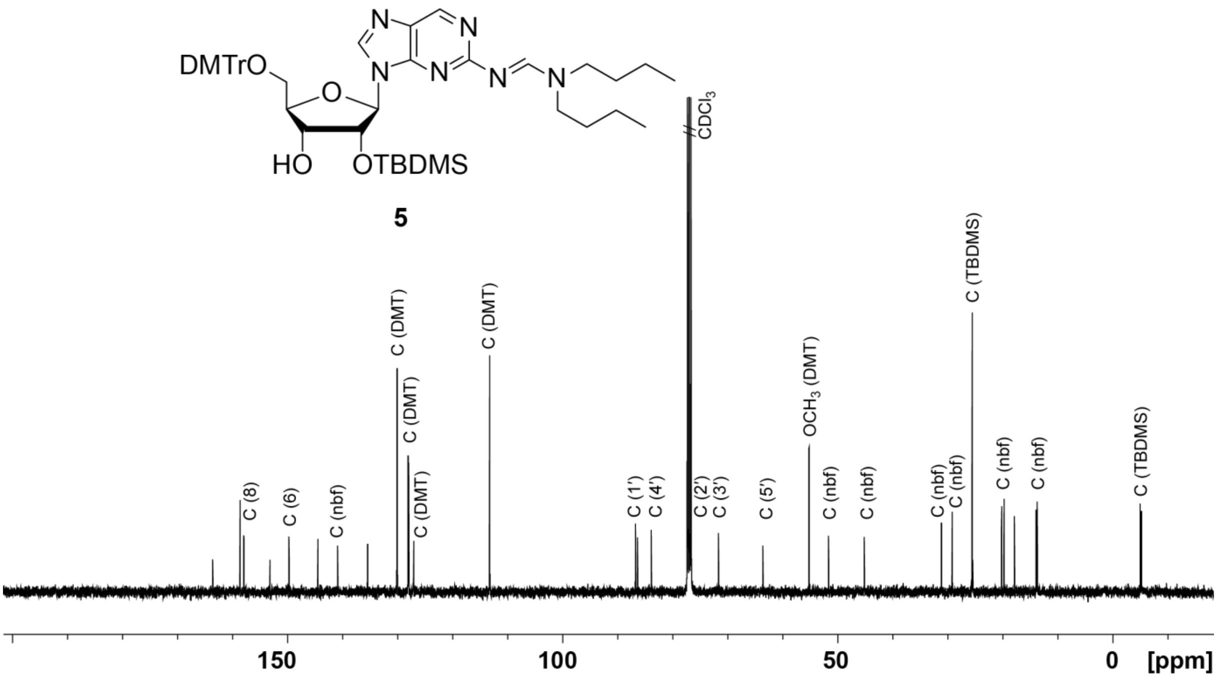

NMR spectra of compound 6

^{1}H-NMR (400 MHz, CDCl$_3$)

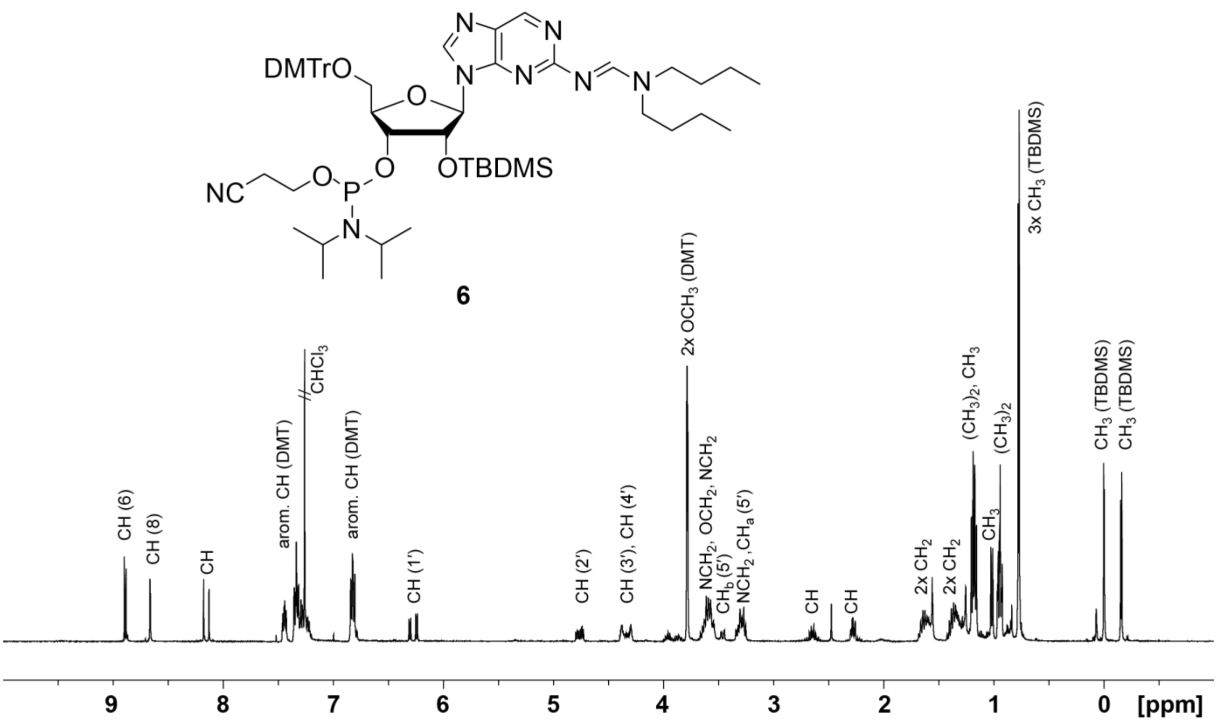

^{13}C-NMR (100 MHz, CDCl$_3$)

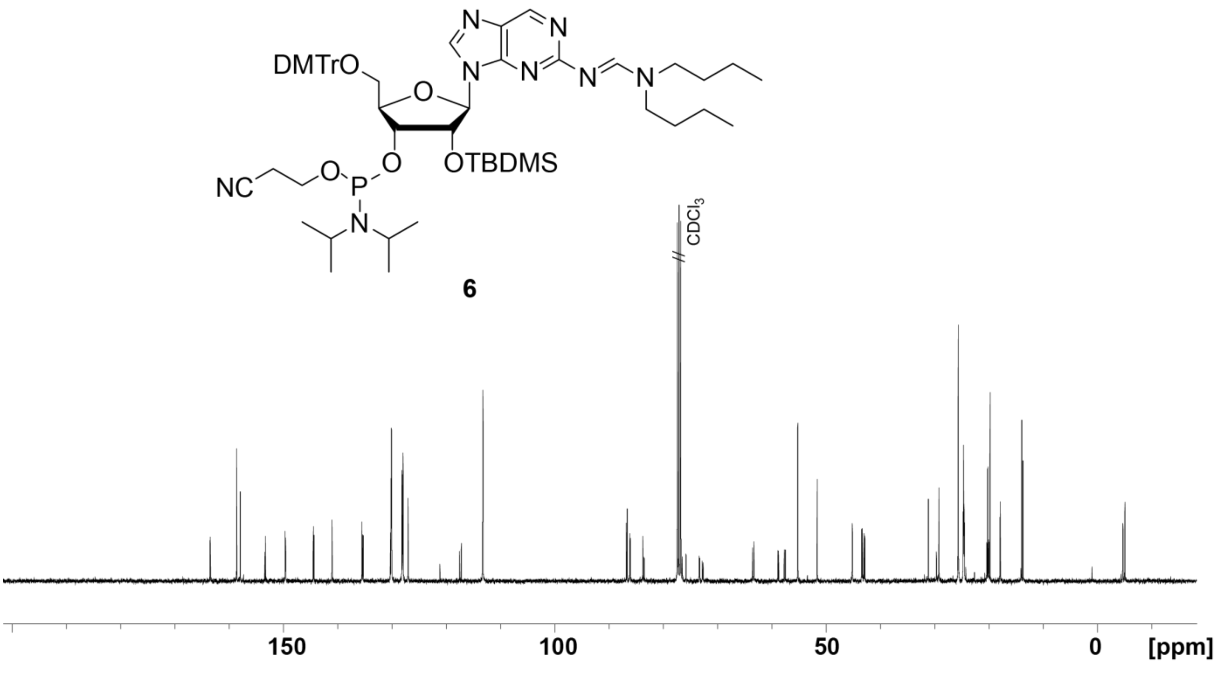

^{31}P-NMR (161 MHz, $CDCl_3$)

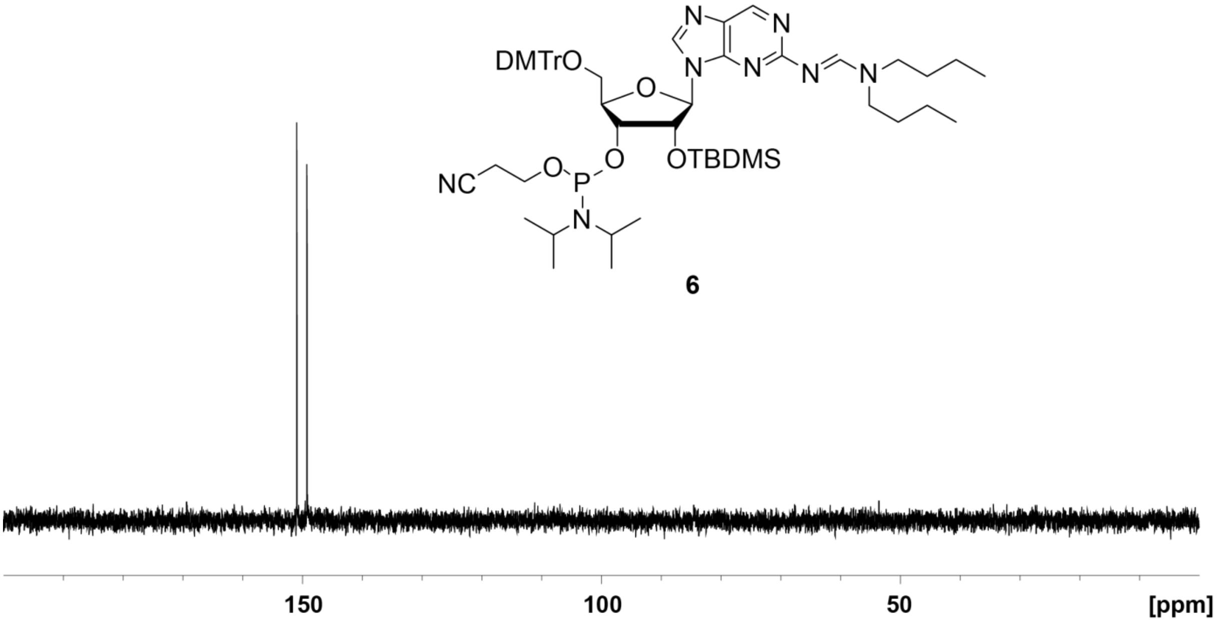

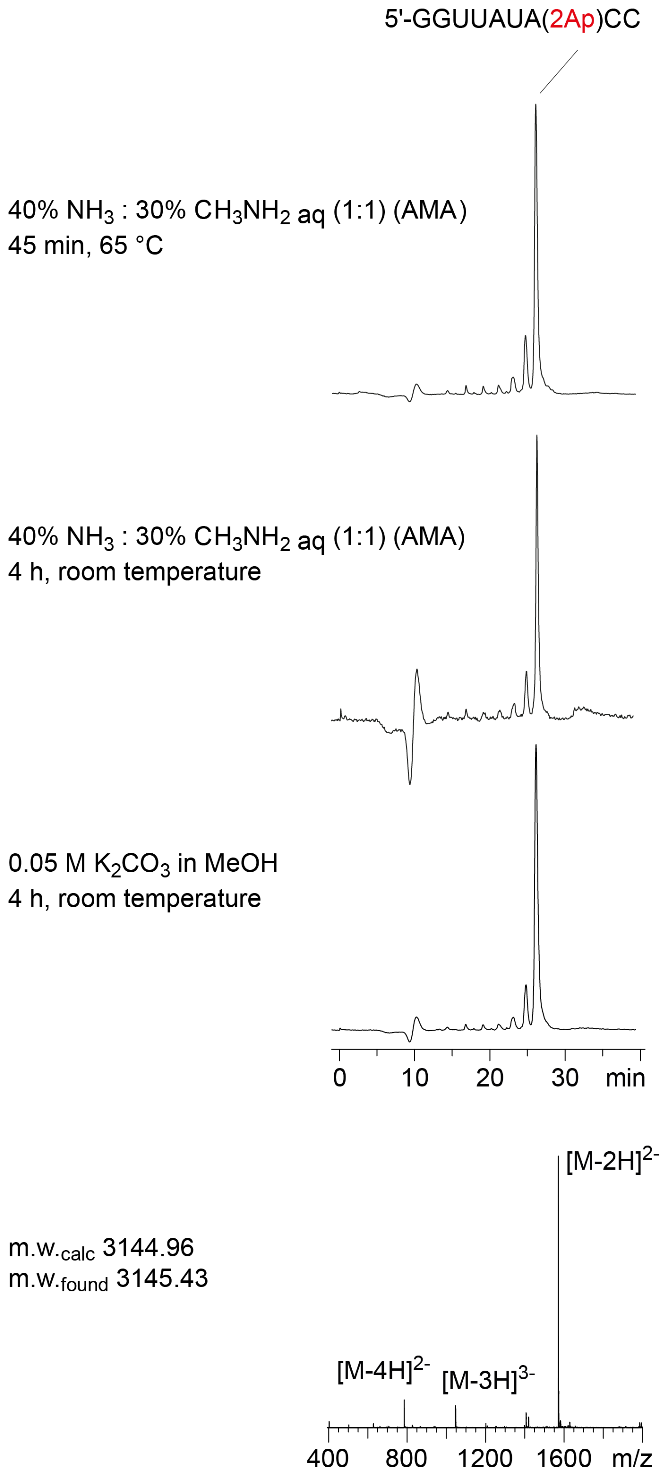

Supporting Figure 1. HPLC analysis of a short Ap-modified RNA deprotected under diverse basic conditions (as indicated). The basic deprotection step was followed by deprotection of the 2'-*O* silyl groups using 1 M TBAF in THF. HPLC conditions: Dionex DNAPac column (4 x 250 mm), 80 °C, 1 mL min^{-1}, 0–60 % buffer B in 45 min. Buffer A: Tris-HCl (25 mm), urea (6 M), pH 8.0. Buffer B: Tris-HCl (25 mm), urea (6 M), $NaClO_4$ (0.5 M), pH 8.0. The lower panel shows the LC-MS spectrum of the purified major product.
